# Functional trajectories associated with acute illness and hospitalization in oldest old patients: Impact on mortality

**DOI:** 10.3389/fphys.2022.937115

**Published:** 2022-09-14

**Authors:** Eva Gallego-González, Jennifer Mayordomo-Cava, María T. Vidán, María I. Valadés-Malagón, José A. Serra-Rexach, Javier Ortiz-Alonso

**Affiliations:** ^1^ Geriatric Department, Hospital General Universitario Gregorio Marañón, Madrid, Spain; ^2^ Instituto de Investigación Sanitaria Gregorio Marañón, Madrid, Spain; ^3^ Hospital Universitario HM Montepríncipe, Madrid, Spain; ^4^ Biomedical Research Networking Center on Frailty and Healthy Aging, CIBERFES, Madrid, Spain; ^5^ School of Medicine, Department of Medicine, Universidad Complutense, Madrid, Spain

**Keywords:** acute care, activities of daily living, functional decline, functional recovery, oldest old patients

## Abstract

**Background:** The literature pays low attention to functional changes during acute illness in older patients. Our main objectives were to separately describe the different functional changes occurring before and after hospital admission in oldest old medical patients, to investigate their association with mortality, and identify predictors associated with in-hospital failure to recover function.

**Methods:** Secondary analysis of data from a prospective cohort study conducted in a tertiary teaching hospital. The study followed the STROBE criteria. The sample included 604 consecutive patients aged 65 or older hospitalized for acute illness, discharged alive, and not fully dependent at baseline. Activities of daily living measured at baseline, admission, and discharge were used to classify patients into four functional trajectories depending on whether they decline or remain stable between baseline and admission (prehospital) and whether they decline, remain stable, or recover baseline function between admission and discharge (in-hospital). Multivariate models were used to test the association between functional trajectories with mortality, and predictors for in-hospital recovery.

**Results:** Functional trajectories were: “stable-stable” (18%); “decline-recovery” (18%); “decline-no recovery” (53%); “in-hospital decline” (11%). Prehospital decline occurred in 75% and 64% were discharged with worse function than baseline. “In-hospital decline” and “decline-no recovery” trajectories were independently associated with higher 6- and 12-month mortality. Extent of prehospital decline and dementia were predictors of failure to in-hospital recovery.

**Conclusion:** In acutely ill older people, differentiating between prehospital and in-hospital functional changes has prognostic implications. Lack of functional regain at discharge is associated with higher mortality at 6- and 12-months.

## Introduction

Functional decline (FD) associated with hospitalization for acute illness is the loss of at least one of the basic activities of daily living (ADLs) at the time of discharge, compared to the onset of the acute illness (Covinsky et al., 2011) has been an important problem in older adults for almost 40 years (Warshaw et al., 1982), and is associated with many deleterious outcomes, including sustained disability, nursing home placement, caregiver burden, and death (Sager et al., 1996b; Covinsky et al., 1997; Inouye et al., 1998; Fortinsky et al., 1999; Rozzini et al., 2005; Boyd et al., 2008; Covinsky et al., 2011; Gallego Gonzalez et al., 2017). Prior studies have shown that functional decline develops in 30%–50% of older patients admitted to the hospital (Warshaw et al., 1982; Hirsch et al., 1990; Inouye et al., 1993; Sager et al., 1996a; Covinsky et al., 2003; Sleiman et al., 2009; Mudge et al., 2010; Zisberg et al., 2015), and the incidence is even higher in frail older adults (Gill et al., 2010).

The trajectories of the functional changes in hospitalized older people are complex. Some patients show a decline at the time of admission, which might persist or recover during hospital stay whereas other patients show a new (or continuing) decline throughout hospitalization (Hirsch et al., 1990; Inouye et al., 1993; Inouye et al., 1998; Fortinsky et al., 1999; Covinsky et al., 2003; Mudge et al., 2010). Prehospital and in-hospital functional changes are different processes, with the former related to the acute illness and the latter reflecting the interaction of illness with hospital care. Previously reported rates of prehospital and in-hospital functional decline show that most of the decline occurs prior to admission with rates varying widely from 29% to 78% (Hirsch et al., 1990; Fortinsky et al., 1999; Covinsky et al., 2003; Sleiman et al., 2009; Mudge et al., 2010; Palleschi et al., 2011; Zaslavsky et al., 2015; Rodrigues et al., 2020), whereas the prevalence of in-hospital decline is usually much lower (5%–32%) (Hirsch et al., 1990; Fortinsky et al., 1999; Covinsky et al., 2003; Mudge et al., 2010; Zaslavsky et al., 2015).

Distinction between prehospital and in-hospital functional changes and their impact on health-related outcomes is important to clinicians, patients, and health administrators. Some studies have examined how prehospital, and in-hospital functional changes influence the risk of mortality (Covinsky et al., 1997; Inouye et al., 1998; Covinsky et al., 2000; Rozzini et al., 2005; Sleiman et al., 2009). It has been shown that baseline functional status, the magnitude of prehospital functional decline and lack of functional regain during hospitalization are associated with 3-, 6-, and 12-months post-discharge mortality. However, little is known regarding the relationship between the different functional trajectories with mortality, more specifically if there are differences in mortality between pre-hospital and in-hospital functional decline. Therefore, to contribute to this topic, the first purpose of our study was to analyze the different functional trajectories occurring before and after hospital admission in a representative group of very old medical patients admitted to the Acute Care for Elders (ACE). The second goal was to assess how the different trajectories influence mortality at 6 and 12 months after discharge.

Most functional decline appears to occur prior to hospitalization and is not preventable by hospital management, suggesting that hospital interventions should be directed to functional recovery among those with prehospital decline. Although risk factors for in-hospital decline have been extensively studied (Sager et al., 1996b; Covinsky et al., 1997; Sleiman et al., 2009; Zaslavsky et al., 2015; Zisberg et al., 2015; Gallego Gonzalez et al., 2017), few studies have examined in-hospital recovery (Boyd et al., 2008; Palleschi et al., 2011; Zaslavsky et al., 2015). Therefore, the third goal of the study was to identify risk factors associated with in-hospital failure to recover in those patients with prehospital decline.

## Methods

### Setting and study population

Prospective observational cohort study of older adults admitted to the ACE Unit of a tertiary teaching hospital according to STROBE criteria (von Elm et al., 2008). The study was approved by the Ethical Committee (No. 39/09). Written informed consent was obtained from all patients/relatives.

**FIGURE 1:**
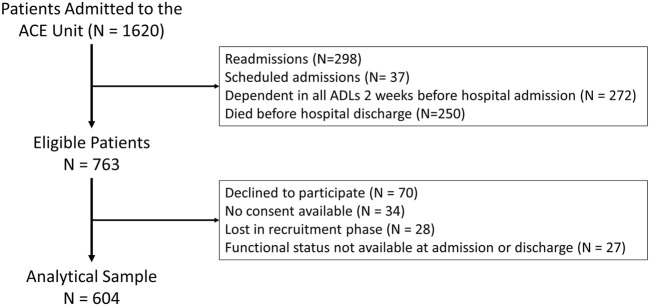
Flow-Chart. Derivation of the study sample.

The inclusion criteria were first time, non-elective admission to the ACE Unit from March 2009 and May 2011 and being discharged alive. The derivation of the study sample is shown in the flow-chart in Figure 1. Patients dependent in all ADLs before hospitalization (N = 272) were excluded because it was not possible to measure additional declines in ADL function. Patients who died before hospital discharge (N = 250) were excluded because did not complete functional trajectories. Functional trajectories were missing in 27 of the eligible patients. Finally, a total of 604 patients were analyzed.

Reason for hospitalization was an acute condition or exacerbation of a chronic one. Most patients were admitted through the Emergency Department (ED). Usual care in the unit included a multicomponent intervention integrated into routine practice that has shown to reduce the incidence of delirium (Vidan et al., 2009) and includes educational measures and specific actions in seven risk areas: orientation, sensory impairment, sleep, mobilization, hydration, nutrition, and drug use. No formal physical therapy was incorporated into the unit.

### Data collection

Data were collected by Geriatric nurses and physicians trained in functional assessment using interviews and medical records at admission (first 48 h), discharge (last 24 h of stay), and by phone at 6 and 12 months after discharge. Baseline assessment took place on admission through interviews with each patient (or a caregiver when the patient was not mentally aware). The patient and patient’s primary nurse were also interviewed at discharge. Medical records were reviewed to ascertain the presence of chronic medical conditions, clinical variables, main admission diagnosis severity of illness, in-hospital complications, and length of hospital stay.

### Outcome measures

#### Functional state: Activities of daily living

Functional status was measured as the total number of ADLs for which the patient was independent, using a modified Katz Index (Katz et al., 1963), which includes bathing, toileting, dressing, transferring and feeding oneself. Continence was not included because medications and standards of care in the hospital setting could make its assessment unreliable. For each ADL a score of 0 was assigned for dependence and 1 for independence. A global summary score was constructed by summing each ADL, ranging from 0 (“completely dependent”) to 5 (“completely independent”). ADLs were assessed at three time points during the study: 1) baseline (two weeks prior to hospitalization, based on patient or caregiver recollection of ADL function), 2) admission, and 3) discharge from the hospital.

### Description of functional trajectories

Patients were classified into one of four mutually exclusive functional trajectories of ADLs depending on whether they declined or remained stable between baseline and admission (prehospital) and whether they declined, remained stable, or recovered baseline function between admission and discharge (in-hospital). We used the term “stable” function when the ADLs score remained constant between two time periods, “decline” when there was a loss of at least one ADL between two time periods, and “recovery” when the patients recovered their baseline function at the time of discharge. The four different trajectories included: 1) prehospital and inhospital stable function (“stable-stable”); 2) prehospital decline but recovered baseline function at discharge (“decline-recovery”); 3) prehospital decline and did no recover to baseline function (“decline-no recovery”); and 4) in-hospital decline, either with or without previous prehospital decline (“in-hospital decline”).

### Mortality

Six- and 12-months post-discharge, mortality was assessed through hospital records or contact with the patient or relatives by telephone interview. If contact with patients was not possible, the local official death records were reviewed. The number of cases censored before the end of the study (left censored) were 4 and 6 for the 6- and 12-month mortality respectively.

### Descriptive variables

Socio-demographic characteristics included age (divided into three different categories), sex, and residence (home or nursing home) before admission. Baseline and admission health and functional status, the ability to walk (dichotomized as independent or dependent as assessed by a Modified Functional Ambulation Classification) (Holden et al., 1984) prior hospital admissions in the last year, nutritional status (with malnutrition defined as the presence of body mass index <23 kg/m2 or albumin concentration in blood <3.5 g/dl) (Espaulella et al., 2007; Bouchard et al., 2009), Charlson index of comorbidities (Charlson et al., 1987), severity of acute illness measured by the APACHE II scale (Knaus et al., 1985), and heart failure severity assessed by the NYHA scale (Fisher, 1972). In-hospital data included main admission diagnosis, length of hospital stay, prevalence of pressure sores, presence of anemia or renal injury at discharge, and incidence of delirium. Delirium was assessed by the chart-based method (Saczynski et al., 2014) and was considered present if the chart review at discharge included any of the following items: agitation, aggressiveness, lethargy, physical restraints, neuroleptics as needed, or diagnosis of delirium in the discharge report.

### Data analysis

Functional trajectories were calculated as defined above and graphically presented. The characteristics of the study participants according to the functional trajectories were organized by descriptive statistics. Differences in demographic, medical and functional measures between functional trajectories were examined using the one-way ANOVA or chi-square test for continuous or categorical variables, respectively. In variables with differences among the means in ANOVA, post-hoc pairwise comparisons were analyzed with Bonferroni correction. Post-hoc tests of categorical variables found significant in overall chi-square analysis, were studied using adjusted standardized residuals with Bonferroni correction (García-pérez and Núñez-antón, 2003).

The predictive value of functional trajectories with 6 and 12-month mortality was assessed. The unadjusted association of risk factors with 6 and 12-month mortality was determined with bivariate Cox proportional hazards regression models. The independent association of functional trajectories and other significant predictors found in the bivariate model was assessed with multivariate Cox models.

The analysis of risk factors associated with in-hospital failure to recover included only patients with prehospital functional decline. Among these patients, differences in demographic, medical, and functional measures between individuals with and without baseline functional recovery at discharge, were examined using a t test or ANOVA for continuous variables, and a chi-square test for categorical variables. Multivariate logistic regression analysis was used to estimate the odds ratio for each of the independent variables. Variables with a *p*-value <0.2 in univariate analysis, and those not significant but considered clinically relevant were entered in the model using backward stepwise (Wald) elimination. Statistical significance was set at a *p*-value of <0.05.

“Missing Values Analysis” was used for descriptive statistics of the missing values and the Little´s chi-square statistic was used for testing whether values are missing completely at random (MCAR). Outcome values on functional status were missing in 27 patients (4.28%). Baseline characteristics or functional status were not related to missing functional status The results of the Little´s missing completely at random (MCAR) test was not significant (Chi-square = 14.977, DF 12, Sig. = .243). Therefore, with a low percent of missing data (<5%), and missing completely at random, we believe that the method of listwise deletion is relatively safe, and we made no imputations. All the analyses were performed with the Statistical Package for Social Science (SPSS), version 28.0.1.1.

## Results

### Characteristics of subjects

Table 1 describes the characteristics, and mortality rate of the 604 patients stratified according to 4 functional trajectories. Mean age was 87 years, the majority were female and admitted from home. The median length of stay was 7 days. At baseline, only 29% were independent in all ADLs and 51% were fully ambulatory. APACHE II score and Charlson index suggested moderate severity of acute illness and moderate levels of comorbidities. Over 50% were malnourished at admission. The overall 6- and 12-months mortality were 22% and 34% respectively. The associations between patient characteristics and functional trajectories are also illustrated in Table 1. Differences between trajectories included ADLs score at admission and discharge, comorbidities (dementia and cerebrovascular disease), incidence of delirium, and 6- and 12-months mortality.

**TABLE 1:** Baseline, In-Hospital Characteristics, and Mortality Rate of the 604 Patients and its Stratification According to Functional Trajectories^a^.

Characteristics	Functional trajectories^b^					
	Total *n* = 604	Stable-stable *n* = 108	Decline-recovery *n* = 107	Decline-no recovery *n* = 319	In-hospital decline *n* = 70	*p* -value
Demographic—*n* (%)						
Age (y)	217 (36)	43 (40)	43 (40)	109 (34)	22 (31)	.798
<85	166 (27)	30 (28)	28 (26)	87 (27)	21 (30)	
85–89	221 (37)	35 (32)	36 (34)	123 (39)	27 (39)	
≥90						
Women	353 (58)	59 (55)	58 (54)	191 (64)	45 (64)	.442
Admitted from NH	67 (11)	10 (9)	9 (8)	44 (11)	4 (6)	.135
Baseline independence in Functional Status—*n* (%)						
ADLs	176 (29)	30 (28)	40 (37)	85 (27)	21 (30)	.203
Ambulation	306 (51)	53 (49)	65 (61)	151 (47)	37 (53)	.111
ADLs score at different time periods—mean ± SD						
Baseline	3.1 ± 1.6	2.8 ± 1.8	3.2 ± 1.8	3.1 ± 1.6	3.3 ± 1.5	.167
Admission	1.4 ± 1.6	3.0 ± 1.8	1.4 ± 1.5	0.6 ± 0.8	2.5 ± 1.5	<.001
Discharge	1.8 ± 1.8	3.1 ± 1.8	3.3 ± 1.8	1.0 ± 1.1	0.9 ± 1.1	<.001
Comorbidities—mean ± SD/*n* (%)						
Charlson Index	2.7 ± 2.0	2.4 ± 1.8	2.4 ± 1.9	2.8 ± 2.1	2.9 ± 2.0	.112
Dementia	178 (30)	15 (14)	26 (24)	121 (38)	16 (23)	<.001
CHF	259 (43)	58 (54)	43 (40)	124 (39)	34 (49)	.037
CBVD disease	137 (23)	15 (14)	19 (18)	89 (28)	14 (20)	.009
Diabetes	173 (29)	29 (27)	29 (27)	87 (27)	28 (40)	.171
COPD	157 (26)	35 (32)	28 (26)	77 (24)	17 (24)	.393
Cancer	84 (14)	11 (10)	18 (17)	46 (14)	9 (13)	.543
Main admission diagnosis—*n* (%)						
Cardiovascular	161 (27)	42 (39)	26 (24)	70 (22)	23 (33)	.004
Respiratory	146 (24	25 (23)	23 (22)	84 (26)	14 (20)	.581
CNS	80 (13)	7 (6)	15 (14)	52 (16)	6 (9)	.041
Renal & Urologic	76 (13)	7 (6)	13 (12)	44 (14)	12 (17)	.143
Admission parameters and in-hospital evolution - mean ± SD/*n* (%)						
APACHE II Score	12.8 ± 4.4	12.9 ± 4.0	12.8 ± 4.6	12.9 ± 4.5	12.6 ± 4.9	.977
Malnutrition	306 (51)	38 (35)	54 (51)	172 (54)	42 (60)	.003
Delirium	220 (36)	24 (22)	36 (34)	137 (43)	23 (33)	<.001
Pressure Sores	57 (10)	7 (7)	4 (4)	38 (12)	8 (11)	.050
Anemia discharge^c^	399 (66)	58 (54)	74 (69)	219 (69)	48 (69)	.030
Length of stay^d^	7 (5–10)	7 (4–9)	7 (5–10)	7 (5–12)	7 (5–9)	.081
Mortality—*n* (%)						
6-month	133 (22)	14 (13)	16 (15)	81 (25)	12 (31)	.003
12-month	207 (34)	27 (25)	28 (26)	121 (38)	31 (44)	.007

a Numbers are mean ± SD or *n* (%) were appropriate.

b Functional trajectories of ADLs: *stable-stable* denotes prehospital and in-hospital stability; *decline-recovery* denotes prehospital decline and in-hospital recovery at discharge; *decline-no recovery* denotes prehospital decline and no recovery at discharge; *in-hospital decline* denotes in-hospital decline, with or without prehospital decline.

c Anemia, defined as hemoglobin <12 g/dl in women and <13 g/dl in men.

d Median length of stay (days; interquartile range).

NH = nursing home; ADLs = Number (%) of patients fully independent in all activities of daily living, including bathing, toileting, dressing, transferring, and feeding oneself; CHF = congestive heart failure; COPD = chronic obstructive pulmonary disease; CNS = central nervous system; Malnutrition refers to the time of admission; CBVD = cerebrovascular disease.

Post-hoc analyses were performed when there were differences in the characteristics of the trajectories (Table 1). A summary of these analyses include: the ADLs at admission were comparable between trajectories Stable-Stable and In-Hospital decline, and both were significantly greater than Decline-No Recovery and Decline-Recovery; the ADLs at discharge were comparable between Stable-Stable and Decline-Recovery and both were greater than In-Hospital Decline and Decline-No recovery. Dementia and cerebrovascular disease were significantly more prevalent in trajectory Decline-No recovery. Malnutrition was less prevalent in Stable-Stable trajectory and the incidence of delirium was greater in Decline-No recovery and lower in Stable-Stable.

### Functional trajectories of patients before and during hospitalization

**FIGURE 2:**
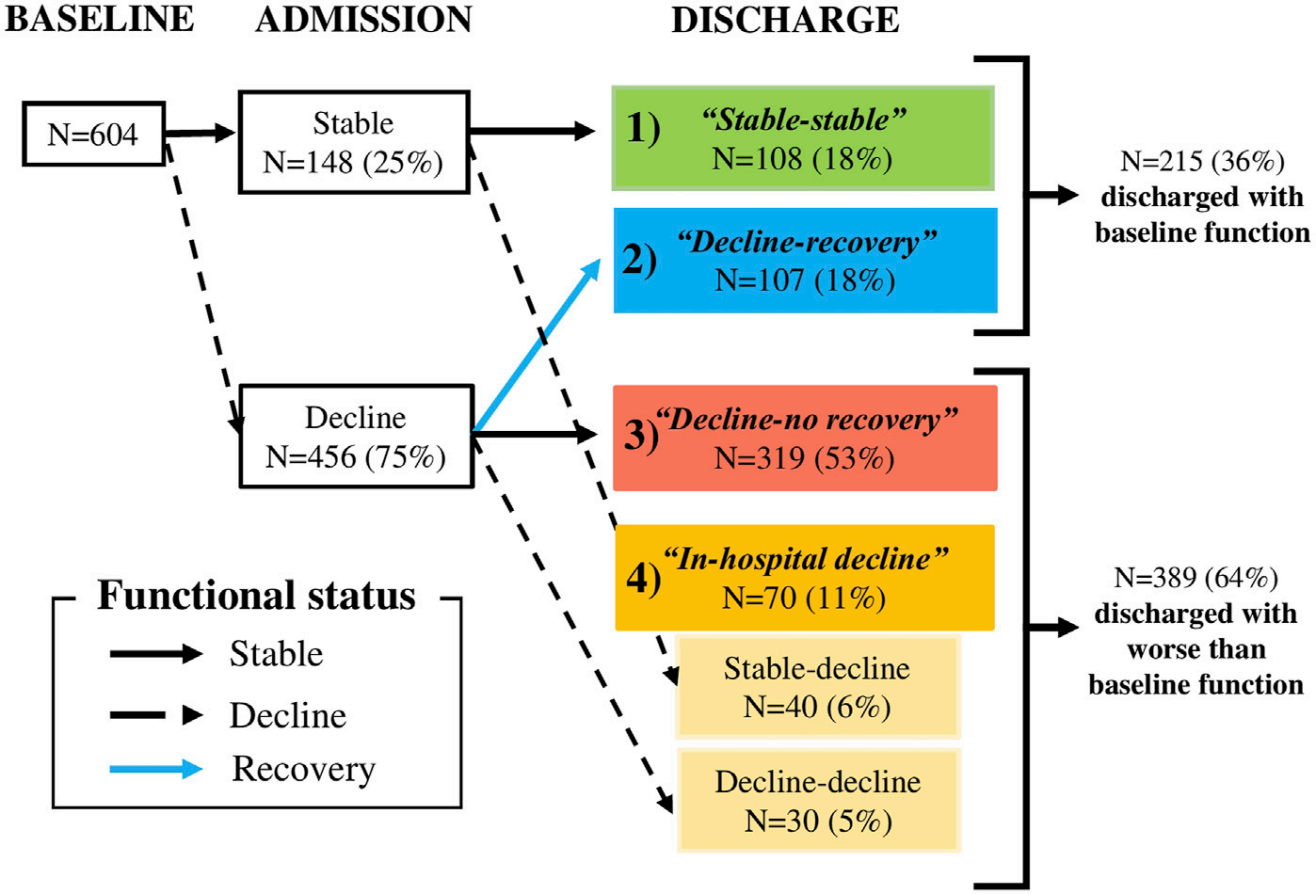
Functional status changes across the three time points of baseline (2 weeks before admission), hospital admission, and discharge. “Stable” refers to the ADLs score remaining constant between two time periods; “decline” refers to the loss of ADLs between two time periods; “recovery” refers to the subjects who recover baseline function at hospital discharge. Boxes at discharge depict the four mutually exclusive functional trajectories of ADLs: 1) “*stable-stable”* denotes prehospital and in-hospital stability, 2) “*decline-recovery”* denotes prehospital decline and in-hospital recovery at discharge, 3) “*decline-no recovery”* denotes prehospital decline and no recovery at discharge, and 4) “*in-hospital decline”* denotes in-hospital decline, with or without prehospital decline. The right side of the figure depicts overall results of the functional trajectories.

The number (proportion) of patients included in each functional trajectory is shown in Figure 2. Prehospital and in-hospital functional decline occurred in 75% and 11% respectively. Overall, 36% were discharged with baseline function, including stable-stable (18%) and decline-recovery (18%) trajectories, and 64% were discharged with worse than baseline function, including in-hospital decline (11%) and decline-no recovery (53%) trajectories. Overall, most patients (82%) had unstable ADL function consisting of decline-recovery, in-hospital decline, and decline-no recovery trajectories. Of the 75% of patients with preadmission decline, only a quarter (23%) recovered baseline function at discharge.

### Functional trajectories and mortality

**FIGURE 3:**
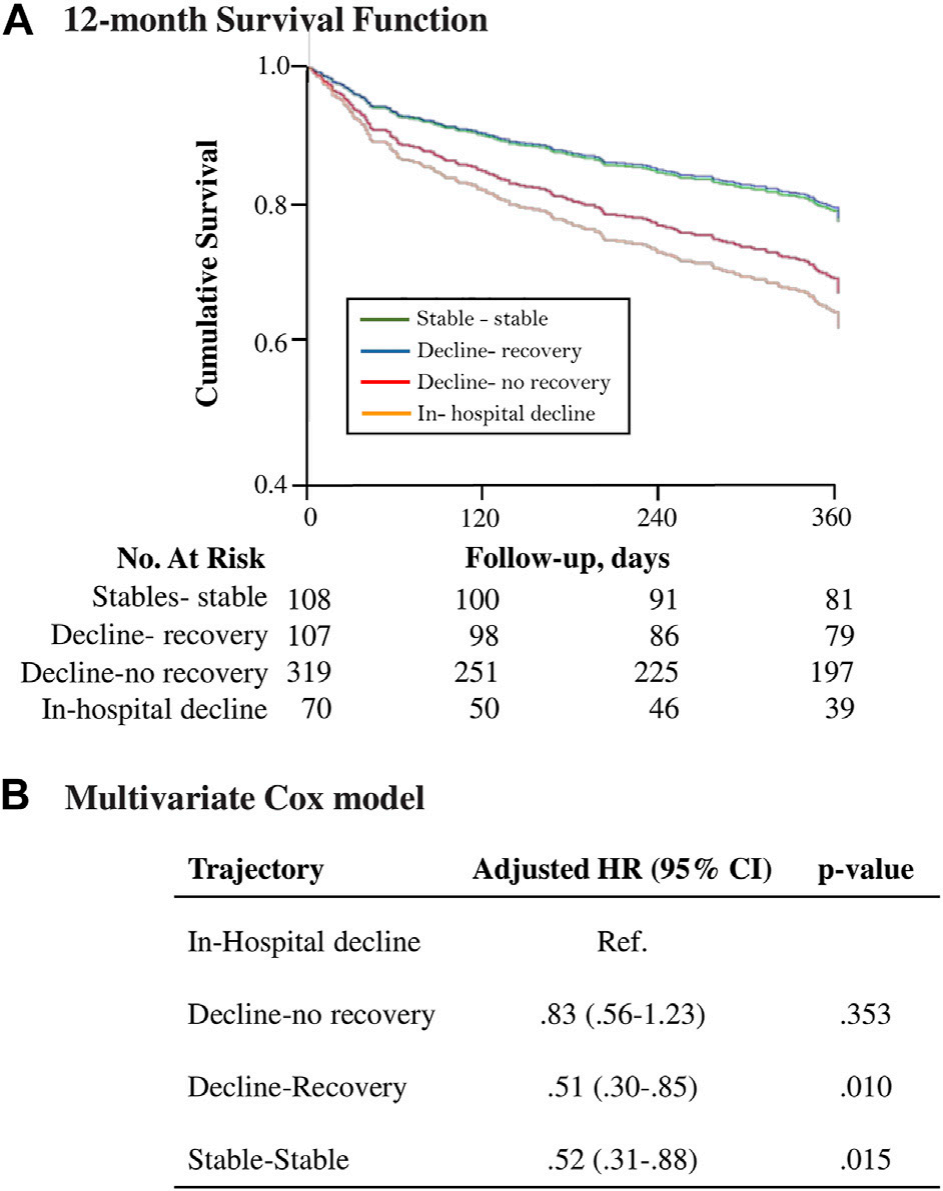
**(A)** Estimated 12-month cumulative survival plot for the 4 functional trajectories, and **(B)** the adjusted hazard ratios (HR) using the “in-hospital” group as the reference category, assessed with multivariate Cox models. Adjusted for age, gender, residence before admission, prior hospital admissions, baseline function, Charlson index, malnutrition, the presence of dementia, and anemia at discharge. Functional status change categories denote prehospital and in-hospital stability (“stable-stable”), prehospital decline and in-hospital recovery at discharge (“decline-recovery”), prehospital decline and no recovery at discharge (“decline-no recovery”), and in-hospital decline, with or without prehospital decline (“in-hospital decline”).

Mortality after 12 months occurred in 34% of patients. Figure 3 shows the estimated 12-month cumulative survival plot for the four functional trajectories, and the adjusted hazard ratios (HR). Patients with decline-recovery trajectory had comparable mortality to patients with stable-stable trajectory The in-hospital decline trajectory showed the highest mortality but comparable to the decline-no recovery trajectory. Mortality after 6 months was of 22%. The unadjusted and adjusted differences in mortality between the four functional trajectories after 6 months, were fully comparable to those after 12-months (Supplementary Figure S1).

### Risk factors associated with in-hospital failure to recover

Of the 456 patients with prehospital decline, the majority (77%) did not recover baseline function at discharge. Differences between patients with and without functional recovery at discharge are shown in Table 2. Factors independently associated with in-hospital failure to recover baseline included baseline dependence in ambulation, the extent of prehospital functional decline, dementia, cerebrovascular disease, and prevalence of pressure sores, as shown in Table 3.

**TABLE 2:** Characteristics of participants with pre-hospital decline (N = 456) according to recovery or failure to recover baseline functional status by hospital discharge.

Characteristics	Recovery of functional status (*n* = 107) *n* (%)	Failure to recover functional status (*n* = 349) *n* (%)	*p*-value
Age (y)	43 (40)	117 (34)	.426
<85	28 (26)	96 (28)	
85–89	36 (34)	136 (38)	
≥90			
Women	58 (54)	211 (60)	.251
Admitted from nursing home	9 (8)	44 (13)	.236
Baseline dependency in at least 1 ADL	67 (63)	252 (72)	.058
Baseline dependence in ambulation	42 (39)	181 (52)	.022
Extent of pre-hospital ADLs decline	81 (76)	179 (51)	<.001
Decline by 1–2 ADLs	26 (24)	170 (49)	
Decline by ≥ 3 ADLs			
Comorbidities			.204
Charlson Index			
≤2	66 (62)	191 (55)	
>2	41 (38	158 (45)	
Dementia	26 (24)	128 (37)	.018
CHF	43 (40)	139 (40)	.947
COPD	28 (26)	85 (24)	.704
Cerebrovascular disease	19 (18)	95 (27)	.048
Diabetes	29 (27)	97 (28)	.889
Cancer	18 (17)	50 (14)	.526
Pressure sores	4 (4)	40 (12)	.018
Main admission diagnosis	26 (24)	81 (23)	
Cardiovascular	23 (22)	90 (26)	.816
Respiratory	15 (14)	53 (15)	.368
Central nervous system	13 (12)	51 (15)	.767
Renal & Urologic			.521
Admission and in-hospital evolution			
APACHE II score	37 (35)	118 (34)	.883
≥15	70 (65)	231 (66)	
<15	54 (51)	188 (54)	.519
Malnutrition	36 (34)	146 (42)	.130
Delirium during hospitalization	74 (69)	239 (69)	.895
Anemia at discharge^a^	4 (4)	450 (12)	.018
Pressure sores	37 (35)	118 (34)	.890
Median length of stay (days)^b^			
≤7	56 (52)	180 (52)	
>7	51 (48)	169 (48)	

a Anemia defined as hemoglobin <12 g/dl in women and <13 g/dl in men.

b Median length of stay (days; interquartile range).

ADLs = Activities of daily living; CHF = congestive heart failure; COPD = chronic obstructive pulmonary disease; CNS = Central nervous system.

**TABLE 3:** Multivariate analysis of factors independently associated with in-hospital failure to recover baseline ADLs function (*n* = 456).

Variable	Adjusted^a^ odds ratio (95% confidence interval)	*p*-value
Baseline dependence in ambulation (yes)	2.47 (1.45–4.22)	<.001
Extent of prehospital decline	Ref	<.001
Loss of 1 or 2 ADLs	5.34 (3.07–9.28)	
Loss of at least 3 ADLs		
Dementia (yes)	1.71 (.99–2.95)	.052
Prevalence of pressure sores (yes)	3.10 (1.05–9.15)	.040
Cerebrovascular disease (yes)	1.86 (1.04–3.33)	.036

a Adjusted for baseline ADLs dependence and the incidence of delirium.

## Discussion

This study contributes to the knowledge of functional changes in “oldest old” patients hospitalized for acute medical illnesses in several ways. First, we found that the majority (82%) had unstable ADL function including high preadmission decline and low in-hospital decline, most (64%) were discharged with worse ADL function than baseline, and less than a quarter of those with preadmission decline recovered baseline function at discharge. Second, functional changes occurring during admission were crucial determinants of 6 and 12-month mortality. Patients with in-hospital decline had the highest risk of mortality, while patients who recovered function during hospitalization had the lowest risk of mortality, comparable to the group with stable function before and during hospitalization. Finally, patients with dementia, poor baseline function, and greater preadmission decline, had a higher risk of in-hospital failure to recover function.

Our reported rates of patients discharged with worse function than baseline (64%) and prehospital decline (75%) were high but consistent with the 28–67% and 38–78% ranges, respectively, that have been previously reported (Hirsch et al., 1990; Fortinsky et al., 1999; Covinsky et al., 2003; Sleiman et al., 2009; Mudge et al., 2010; Palleschi et al., 2011; Zaslavsky et al., 2015; Rodrigues et al., 2020) and might be related to the very old age and poor baseline functional status. The low rate of in-hospital decline (11%) was consistent with the 7%–28% reported previously (Hirsch et al., 1990; Fortinsky et al., 1999; Covinsky et al., 2003; Mudge et al., 2010; Zaslavsky et al., 2015), and might be related to the usual care in our ACE unit (Vidan et al., 2009) and the exclusion of 19% of the eligible patients that died during the hospitalization, In our study, the rate of in-hospital recovery after preadmission decline (23%), was lower than the 42–66% reported rates of most studies (Covinsky et al., 2003; Mudge et al., 2010; Palleschi et al., 2011). These differences of in-hospital recovery of function lost prior to admission with other studies, might be related to the frail population in our study as suggested by the findings that dementia, cerebrovascular disease, baseline dependence in ambulation, and extent of prehospital ADLs decline were related to failure to recover baseline function, or to differences in health care systems and access to rehabilitation during hospitalization.

Functional changes during acute illness and hospitalization contain important information about risk of mortality. In our study, functional trajectories remained an independent factor associated with mortality. Our results extend those of previous studies demonstrating that patients who recovered baseline function at discharge had the lowest mortality (Rozzini et al., 2005; Sleiman et al., 2009). Patients with in-hospital decline (with or without preadmission decline) were associated with the highest mortality, suggesting that during an acute illness, the best prognostic changes are those in which the patients are able to maintain or regain baseline function, and the worse prognostic changes are those discharged with worse function than baseline. Furthermore, disability acquired in the hospital is a contributor to death as strong as the failure to recover function lost before admission. Therefore, assessing a patient’s functional status at baseline, admission, and discharge from the hospital may have a prognostic sign beyond the value provided by clinical or laboratory data.

Our study extends previous data regarding predictors of in-hospital failure to recover baseline function at discharge (Covinsky et al., 2003; Lindenberger et al., 2003; Mudge et al., 2010). In addition to the presence of dementia and the extent of prehospital decline (Mudge et al., 2010), we found that the prevalence of pressure sores and cerebrovascular disease were also associated with failure to recover. Although the extent of prehospital decline is likely to represent the effects of acute illness, we found no association of functional recovery with severity of acute illness as measured by the APACHE II score. In this regard, it might be possible that the extent of prehospital functional decline is a better marker of clinical instability (Covinsky et al., 1997; Inouye et al., 1998). Contrary to other studies (Covinsky et al., 2003; Mudge et al., 2010), we found no association of age with failure to recover function at discharge. However, in the study of Covinsky et al. (2003), age was associated with functional changes after, but not before, admission. In our study the absence of effects of age is consistent with the finding that age did not affect adverse functional change before or after admission, or baseline degree of dependency. We believe this is the study with the highest proportion of persons older than 85 years (64%). As suggested previously (Hamerman, 1999; Rozzini et al., 2000; Covinsky et al., 2003; Rozzini et al., 2005), it is possible that, in this “oldest old” population the inability to regain function after an acute illness might be a consequence of frailty.

Several study limitations are noteworthy. First, a systematic bias due to rater report of baseline functional status may partly explain the high rate of prehospital functional decline. Rubenstein et al. (1984) noted that patients tend to rate their level of functioning higher than nurses. In this study the preadmission assessments came from patients or caregivers and the in-hospital assessment from nurses. This may lead to greater apparent preadmission decline. However, Covinsky et al. (2000) found that the baseline assessment at the time of hospital admission, has face and predictive validity. Second, functional evaluation of the individual ADLs was based on the Katz index with a dichotomous response, indicating complete independence or not. It is possible that no changes were detected in individual ADLs recovery because the scale lacks sensitivity to detect subtle changes over time. Third, length of stay is a potential confounding that may contribute to discrepancies in rates of functional decline (Pedone et al., 2005; Volpato et al., 2007). However, in this study the length of stay was similar in the 4 functional trajectories. Fourth, it was not possible to consider all predictors of functional lack of recovery, included depression (Shahab et al., 2017) and family and caregiver factors (Boltz et al., 2018), therefore, the impact of them could not be evaluated. Finally, this is a single-site study that was limited to the healthiest survivors; consequently, the findings may not apply to all patients admitted to an ACE unit.

## Conclusion and implications

This study provides unique data regarding functional changes in a representative group of very old medical patients admitted to an ACE unit. In this study we found that functional changes are very common, and most patients were discharged with worse function than their preadmission level. The decline occurred most before hospital admission, with a low rate of in-hospital decline, and a low rate of in-hospital recovery in those with preadmission decline.

Our data add to the knowledge of how preadmission and in-hospital decline are related to mortality, highlights the need to monitor the functional status of hospitalized older people, and may be useful for counseling patients for future care. For patients who have lost independence in functional status at admission, rehabilitation should be a goal of care to regain independence at discharge.

In this oldest old population, the inability to remain functionally stable or regain function after an acute illness, may reflect a state of inability to react to stressful events. Differences in the prevalence of in-hospital functional trajectories between health care systems may be related to differences in the age and frailty status of patients admitted to the ACE units. Comparisons of studies from different heath care systems may need to adjust to these potential confounders.

## Data Availability

The raw data supporting the conclusions of this article will be made available by the authors, without undue reservation.

## References

[B1] Boltz M. Lee K. H. Chippendale T. Trotta R. L. (2018). Pre-admission functional decline in hospitalized persons with dementia: The influence of family caregiver factors. Arch. Gerontol. Geriatr. 74, 49–54. 10.1016/j.archger.2017.09.006 28957688

[B2] Bouchard D. R. Dionne I. J. Brochu M. (2009). Sarcopenic/obesity and physical capacity in older men and women: Data from the nutrition as a determinant of successful aging (NuAge)-the quebec longitudinal study. Obes. (Silver Spring) 17 (11), 2082–2088. 10.1038/oby.2009.109 19373219

[B3] Boyd C. M. Landefeld C. S. Counsell S. R. Palmer R. M. Fortinsky R. H. Kresevic D. (2008). Recovery of activities of daily living in older adults after hospitalization for acute medical illness. J. Am. Geriatr. Soc. 56 (12), 2171–2179. 10.1111/j.1532-5415.2008.02023.x 19093915 PMC2717728

[B4] Charlson M. E. Pompei P. Ales K. L. MacKenzie C. R. (1987). A new method of classifying prognostic comorbidity in longitudinal studies: Development and validation. J. Chronic Dis. 40 (5), 373–383. 10.1016/0021-9681(87)90171-8 3558716

[B5] Covinsky K. E. Justice A. C. Rosenthal G. E. Palmer R. M. Landefeld C. S. (1997). Measuring prognosis and case mix in hospitalized elders. The importance of functional status. J. Gen. Intern. Med. 12 (4), 203–208. 10.1046/j.1525-1497.1997.012004203.x 9127223 PMC1497092

[B6] Covinsky K. E. Palmer R. M. Counsell S. R. Pine Z. M. Walter L. C. Chren M. M. (2000). Functional status before hospitalization in acutely ill older adults: Validity and clinical importance of retrospective reports. J. Am. Geriatr. Soc. 48 (2), 164–169. 10.1111/j.1532-5415.2000.tb03907.x 10682945

[B7] Covinsky K. E. Palmer R. M. Fortinsky R. H. Counsell S. R. Stewart A. L. Kresevic D. (2003). Loss of independence in activities of daily living in older adults hospitalized with medical illnesses: Increased vulnerability with age. J. Am. Geriatr. Soc. 51 (4), 451–458. 10.1046/j.1532-5415.2003.51152.x 12657063

[B8] Covinsky K. E. Pierluissi E. Johnston C. B. (2011). Hospitalization-associated disability: "She was probably able to ambulate, but I'm not sure. JAMA 306 (16), 1782–1793. 10.1001/jama.2011.1556 22028354

[B9] Espaulella J. Arnau A. Cubi D. Amblas J. Yanez A. (2007). Time-dependent prognostic factors of 6-month mortality in frail elderly patients admitted to post-acute care. Age Ageing 36 (4), 407–413. 10.1093/ageing/afm033 17395620

[B10] Fisher J. D. (1972). New York heart association classification. Arch. Intern. Med. 129 (5), 836. 10.1001/archinte.1972.00320050160023 5025908

[B11] Fortinsky R. H. Covinsky K. E. Palmer R. M. Landefeld C. S. (1999). Effects of functional status changes before and during hospitalization on nursing home admission of older adults. J. Gerontol. A Biol. Sci. Med. Sci. 54 (10), M521–M526. 10.1093/gerona/54.10.m521 10568535

[B12] Gallego Gonzalez E. Ortiz Alonso F. J. Vidan Astiz M. T. Soria Felix S. Garcia Cardenas V. Omonte Guzman J. (2017). Development and validation of a prognostic index for 6- and 12-month mortality in hospitalized older adults. Arch. Gerontol. Geriatr. 73, 269–278. 10.1016/j.archger.2017.07.008 28869885

[B13] García-pérez M. A. Núñez-antón V. (2003). Cellwise residual analysis in two-way contingency tables. Educ. Psychol. Meas. 63 (5), 825–839. 10.1177/0013164403251280

[B14] Gill T. M. Allore H. G. Gahbauer E. A. Murphy T. E. (2010). Change in disability after hospitalization or restricted activity in older persons. JAMA 304 (17), 1919–1928. 10.1001/jama.2010.1568 21045098 PMC3124926

[B15] Hamerman D. (1999). Toward an understanding of frailty. Ann. Intern. Med. 130 (11), 945–950. 10.7326/0003-4819-130-11-199906010-00022 10375351

[B16] Hirsch C. H. Sommers L. Olsen A. Mullen L. Winograd C. H. (1990). The natural history of functional morbidity in hospitalized older patients. J. Am. Geriatr. Soc. 38 (12), 1296–1303. 10.1111/j.1532-5415.1990.tb03451.x 2123911

[B17] Holden M. K. Gill K. M. Magliozzi M. R. Nathan J. Piehl-Baker L. (1984). Clinical gait assessment in the neurologically impaired. Reliability and meaningfulness. Phys. Ther. 64 (1), 35–40. 10.1093/ptj/64.1.35 6691052

[B18] Inouye S. K. Peduzzi P. N. Robison J. T. Hughes J. S. Horwitz R. I. Concato J. (1998). Importance of functional measures in predicting mortality among older hospitalized patients. JAMA 279 (15), 1187–1193. 10.1001/jama.279.15.1187 9555758

[B19] Inouye S. K. Viscoli C. M. Horwitz R. I. Hurst L. D. Tinetti M. E. (1993). A predictive model for delirium in hospitalized elderly medical patients based on admission characteristics. Ann. Intern. Med. 119 (6), 474–481. 10.7326/0003-4819-119-6-199309150-00005 8357112

[B20] Katz S. Ford A. B. Moskowitz R. W. Jackson B. A. Jaffe M. W. (1963). Studies of illness in the aged. The index of adl: A standardized measure of biological and psychosocial function. JAMA 185 (12), 914–919. 10.1001/jama.1963.03060120024016 14044222

[B21] Knaus W. A. Draper E. A. Wagner D. P. Zimmerman J. E. (1985). Apache II: A severity of disease classification system. Crit. Care Med. 13 (10), 818–829. 10.1097/00003246-198510000-00009 3928249

[B22] Lindenberger E. C. Landefeld C. S. Sands L. P. Counsell S. R. Fortinsky R. H. Palmer R. M. (2003). Unsteadiness reported by older hospitalized patients predicts functional decline. J. Am. Geriatr. Soc. 51 (5), 621–626. 10.1034/j.1600-0579.2003.00205.x 12752836

[B23] Mudge A. M. O'Rourke P. Denaro C. P. (2010). Timing and risk factors for functional changes associated with medical hospitalization in older patients. J. Gerontol. A Biol. Sci. Med. Sci. 65 (8), 866–872. 10.1093/gerona/glq069 20494952

[B24] Palleschi L. De Alfieri W. Salani B. Fimognari F. L. Marsilii A. Pierantozzi A. (2011). Functional recovery of elderly patients hospitalized in geriatric and general medicine units. The PROgetto DImissioni in GEriatria Study. J. Am. Geriatr. Soc. 59 (2), 193–199. 10.1111/j.1532-5415.2010.03239.x 21288230

[B25] Pedone C. Ercolani S. Catani M. Maggio D. Ruggiero C. Quartesan R. (2005). Elderly patients with cognitive impairment have a high risk for functional decline during hospitalization: The GIFA Study. J. Gerontol. A Biol. Sci. Med. Sci. 60 (12), 1576–1580. 10.1093/gerona/60.12.1576 16424291

[B26] Rodrigues C. Mendonca D. Martins M. M. (2020). Functional trajectories of older acute medical inpatients. Enferm. Clin. 30 (4), 260–268. 10.1016/j.enfcli.2019.03.001 31076259

[B27] Rozzini R. Frisoni G. B. Franzoni S. Trabucchi M. (2000). Change in functional status during hospitalization in older adults: A geriatric concept of frailty. J. Am. Geriatr. Soc. 48 (8), 1024–1025. 10.1111/j.1532-5415.2000.tb06911.x 10968318

[B28] Rozzini R. Sabatini T. Cassinadri A. Boffelli S. Ferri M. Barbisoni P. (2005). Relationship between functional loss before hospital admission and mortality in elderly persons with medical illness. J. Gerontol. A Biol. Sci. Med. Sci. 60 (9), 1180–1183. 10.1093/gerona/60.9.1180 16183960

[B29] Rubenstein L. Z. Schairer C. Wieland G. D. Kane R. (1984). Systematic biases in functional status assessment of elderly adults: Effects of different data sources. J. Gerontol. 39 (6), 686–691. 10.1093/geronj/39.6.686 6436360

[B30] Saczynski J. S. Kosar C. M. Xu G. Puelle M. R. Schmitt E. Jones R. N. (2014). A tale of two methods: Chart and interview methods for identifying delirium. J. Am. Geriatr. Soc. 62 (3), 518–524. 10.1111/jgs.12684 24512042 PMC3959564

[B31] Sager M. A. Franke T. Inouye S. K. Landefeld C. S. Morgan T. M. Rudberg M. A. (1996a). Functional outcomes of acute medical illness and hospitalization in older persons. Arch. Intern. Med. 156 (6), 645–652. 10.1001/archinte.1996.00440060067008 8629876

[B32] Sager M. A. Rudberg M. A. Jalaluddin M. Franke T. Inouye S. K. Landefeld C. S. (1996b). Hospital admission risk profile (HARP): Identifying older patients at risk for functional decline following acute medical illness and hospitalization. J. Am. Geriatr. Soc. 44 (3), 251–257. 10.1111/j.1532-5415.1996.tb00910.x 8600192

[B33] Shahab S. Nicolici D. F. Tang A. Katz P. Mah L. (2017). Depression predicts functional outcome in geriatric inpatient rehabilitation. Arch. Phys. Med. Rehabil. 98 (3), 500–507. 10.1016/j.apmr.2016.07.014 27530770

[B34] Sleiman I. Rozzini R. Barbisoni P. Morandi A. Ricci A. Giordano A. (2009). Functional trajectories during hospitalization: A prognostic sign for elderly patients. J. Gerontol. A Biol. Sci. Med. Sci. 64 (6), 659–663. 10.1093/gerona/glp015 19270181

[B35] Vidan M. T. Sanchez E. Alonso M. Montero B. Ortiz J. Serra J. A. (2009). An intervention integrated into daily clinical practice reduces the incidence of delirium during hospitalization in elderly patients. J. Am. Geriatr. Soc. 57 (11), 2029–2036. 10.1111/j.1532-5415.2009.02485.x 19754498

[B36] Volpato S. Onder G. Cavalieri M. Guerra G. Sioulis F. Maraldi C. (2007). Characteristics of nondisabled older patients developing new disability associated with medical illnesses and hospitalization. J. Gen. Intern. Med. 22 (5), 668–674. 10.1007/s11606-007-0152-1 17443376 PMC1852921

[B37] von Elm E. Altman D. G. Egger M. Pocock S. J. Gøtzsche P. C. Vandenbroucke J. P. (2008). The strengthening the reporting of observational studies in epidemiology (STROBE) statement: Guidelines for reporting observational studies. J. Clin. Epidemiol. 61 (4), 344–349. 10.1016/j.jclinepi.2007.11.008 18313558

[B38] Warshaw G. A. Moore J. T. Friedman S. W. Currie C. T. Kennie D. C. Kane W. J. (1982). Functional disability in the hospitalized elderly. JAMA 248 (7), 847–850. 10.1001/jama.1982.03330070035026 6212699

[B39] Zaslavsky O. Zisberg A. Shadmi E. (2015). Impact of functional change before and during hospitalization on functional recovery 1 month following hospitalization. J. Gerontol. A Biol. Sci. Med. Sci. 70 (3), 381–386. 10.1093/gerona/glu168 25199914

[B40] Zisberg A. Shadmi E. Gur-Yaish N. Tonkikh O. Sinoff G. (2015). Hospital-associated functional decline: The role of hospitalization processes beyond individual risk factors. J. Am. Geriatr. Soc. 63 (1), 55–62. 10.1111/jgs.13193 25597557

