# Supplementary material for: Functional trajectories associated with acute illness and hospitalization in oldest old patients: Impact on mortality
Source: Front Physiol. 2022 Sep 14;13:937115. doi: 10.3389/fphys.2022.937115 (PMC9515786; doi:10.3389/fphys.2022.937115)
Supplement: Supplementary file 1 [file Image1.pdf]

A) 6-month Survival Function

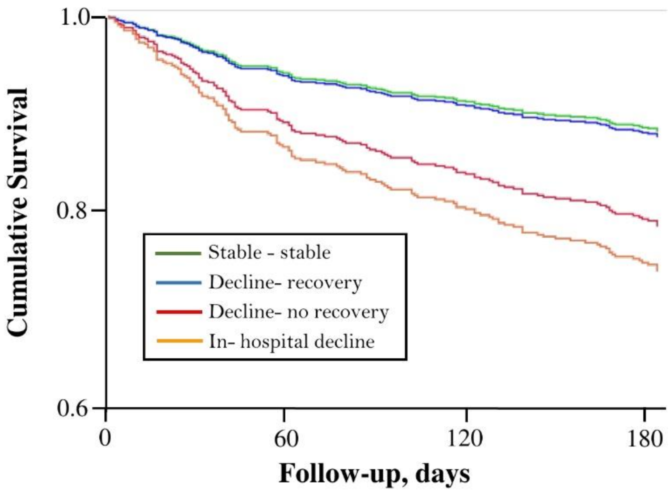

B) Multivariate Cox model

Trajectory	Adjusted HR (95% CI)	p-value
In-Hospital decline	Ref.	
Decline-No recovery	.8 (.50-1.29)	.353
Decline-Recovery	.44 (.23-.83)	.012
Stable-Stable	.42 (.21-.82)	.012

Supplementary Fig. 1. A) Estimated 6-month cumulative survival plot for the 4 functional trajectories, and B) the adjusted hazard ratios (HR) using the "in-hospital" group as the reference category, assessed with multivariate Cox models. Adjusted for age, gender, residence before admission, prior hospital admissions, baseline function, Charlson index, malnutrition, the presence of dementia, and anemia at discharge. Functional status change categories denote prehospital and in-hospital stability ("stable-stable"), prehospital decline and in-hospital recovery at discharge ("decline-recovery"), prehospital decline and no recovery at discharge ("decline-no recovery"), and in-hospital decline, with or without prehospital decline ("in-hospital decline").
